# Transient receptor potential vanilloid subtype 1: A potential therapeutic target for fibrotic diseases

**DOI:** 10.3389/fphys.2022.951980

**Published:** 2022-08-15

**Authors:** Guangxin Peng, Xiaoling Tang, Yang Gui, Jing Yang, Lifang Ye, Liuyang Wu, Ya hui Ding, Lihong Wang

**Affiliations:** ^1^ Zhejiang University of Technology, Hangzhou, China; ^2^ Department of Cardiovascular Medicine, Zhejiang Provincial People’s Hospital, People’s Hospital of Hangzhou Medical College, Hangzhou, China

**Keywords:** fibrotic diseases, transient receptor potential vanilloid type 1, signaling pathways, therapeutic target, neurogenic inflammation

## Abstract

The transient receptor potential vanilloid subtype 1 (TRPV1), belonging to the TRPV channel family, is a non-selective, calcium-dependent, cation channel implicated in several pathophysiological processes. Collagen, an extracellular matrix component, can accumulate under pathological conditions and may lead to the destruction of tissue structure, organ dysfunction, and organ failure. Increasing evidence indicates that TRPV1 plays a role in the development and occurrence of fibrotic diseases, including myocardial, renal, pancreatic, and corneal fibrosis. However, the mechanism by which TRPV1 regulates fibrosis remains unclear. This review highlights the comprehensive role played by TRPV1 in regulating pro-fibrotic processes, the potential of TRPV1 as a therapeutic target in fibrotic diseases, as well as the different signaling pathways associated with TRPV1 and fibrosis.

## Introduction

Fibrosis is a pathophysiological feature primarily characterized by the accumulation of inflammatory cells; increased fibroblast proliferation, migration, and capacity to adhere and deposit extracellular matrix (ECM); and abnormal deposition of collagen-based ECM (Dees et al., 2020). The morbidity and mortality rates of fibrotic diseases remain high. Fibrosis, which is also an aging-related complication, is implicated in numerous chronic diseases; moreover, it can afflict almost any organ or tissue. Diseases associated with fibrosis include liver, lung, and renal diseases, myocardial fibrosis, systemic sclerosis, or any other disease in which, fibrotic remodeling leads to the destruction of organ structure and loss of function (Zhang and Zhang, 2020). Mounting evidence suggests that myofibroblasts play an instrumental role in ECM remodeling and fibrosis (Yazdani et al., 2017). Furthermore, fibroblasts are responsible for the production and early tensing of collagen and play key roles in fibrosis and wound healing. Smooth muscle actin (α-SMA), expressed in differentiated fibroblasts (i.e., myofibroblasts), increases the expression of type I and type III collagen, and inhibits the degradation of ECM macromolecules (Li et al., 2020). The onset and progression of fibrotic disease are associated with complex pathophysiological and biochemical processes regulated by a network of cytokines and intracellular signaling pathways (Henderson et al., 2020). However, identifying putative anti-fibrotic targets and formulating effective therapies for treating fibrotic diseases remain challenging. Transient receptor potential vanilloid (TRPV) channels are essential for various cellular functions and are involved in signaling pathways related to apoptosis, cell proliferation, cell migration, and invasion (Santoni et al., 2011). TRPV subtype 1 (TRPV1) is a member of the TRPV family, and mechanisms underlying its function are relatively well-understood. The TRPV1 receptor is highly permeable to Ca^2+^ and plays a critical role in maintaining Ca^2+^ levels in the cells (Zhai et al., 2020). In addition, calcium-dependent mechanisms are believed to play a crucial role in fibrogenesis *via* transient receptor potential channels (Inoue et al., 2019). Angiotensin II significantly increased the proliferation of cardiac fibroblasts, whereas activation of TRPV1 by capsaicin abolished the proliferation, differentiation and apoptosis of fibroblasts isolated from WT mice, but not in fibroblasts isolated from *TRPV1* ^*-/-*^ mice (Wang H.J et al., 2014). In recent years, a TRPV1 has been strong associated with fibrosis, and has been implicated in fibrotic diseases of various organs. For instance, TRPV1 is considered to regulate renal, lung, and cardiac disease and corneal fibrosis (Wang et al., 2008; Hutchinson et al., 2017; Inoue et al., 2019; Turan et al., 2021). Given the commonalities among fibrotic diseases, summarizing the direct or indirect relationship between TRPV1 and fibrosis as well as elucidating the novel role of TRPV1 in fibrosis will have far-reaching implications for future studies investigating fibrosis onset and development, pathogenesis, and potential novel therapies.

## Overview of TRPV1

TRPV1, a capsaicin receptor first cloned in 1997 (Caterina et al., 1997), is a non-selective cation channel that allows the passage of cations such as H^+^, Na^+^, Ca^2+^, and Mg^2+^, with particularly high permeability for Ca^2+^ (Pedersen et al., 2005). TRPV1 is composed of 838 amino acids with a molecular weight of 95 kDa. The protein has a tetramer structure composed of six hydrophobic groups across the membrane and between the fifth and sixth transmembrane regions. The C- and N-termini of TRPV1 are located inside the cell membrane and regulate its functional activity (Liao et al., 2013). TRPV1 is a multimodal nociceptor that can be activated by a variety of stimuli, including capsaicin, lipopolysaccharide, low osmolarity, high temperature (>43 C), low pH (<6.0), and endogenous or synthetic ligands (Gavva et al., 2008; Gao et al., 2014). Although TRPV1 receptors are widely expressed throughout the nervous system, they are also found in other tissues and organs, including the heart, pancreas, lungs, trachea, kidneys, skin, intestines, and bladder (Faisy et al., 2007; Suri and Szallasi, 2008; Ditting et al., 2012; Thomas et al., 2012; Grundy et al., 2018; Cohen et al., 2019; Yoshie et al., 2020; Deng et al., 2021). At the cellular level, TRPV1 is expressed in lymphocytes, macrophages, dendritic cells, mast cells, endothelial cells, vascular smooth muscle cells, Epithelial cells, and fibroblasts (O'Connell et al., 2005; Kim et al., 2006; Razavi et al., 2006; Tóth et al., 2018; Otto et al., 2020; Luo et al., 2021; Shamsi et al., 2021). TRPV1 channels facilitate Ca^2+^ passage and thus, are involved in calcium signaling that regulates cellular processes related to proliferation, survival, migration, embryonic development, apoptosis, autophagy, and tumor cell metastasis (Waning et al., 2007; Takahashi et al., 2014; Malenczyk et al., 2017; Xu et al., 2018; Nakazawa et al., 2019; Chen et al., 2021; Güzel and Akpınar, 2021). Following TRPV1 channel activation, large amounts of calcium ions flow inward, while some potassium ions flow outward, thereby stimulating nerve endings to generate action potential and undergo depolarization. This activates a series of intracellular signals and the release of neurotransmitters such as calcitonin gene-related peptide (CGRP), substance P (SP), and somatostatin (SOM, a growth inhibitory hormone), thus, triggering multiple signaling cascades (Holzer, 1988). Most of the research studies investigating the functions of the TRPV1 channel have focused on screening TRPV1 agonists and antagonists and evaluating their role in TRPV1-related diseases and associated mechanisms. The exogenous agonists of TRPV1 include capsaicin, resin toxin, piperine, wogonin, norepinephrine, and cannabinol. The endogenous agonists of TRPV1 include cannabinoids, *N*-arachidonic ethanolamine, leukotriene B4, *N*-arachidonic dopamine, palmitoylethanolamide, and *N*-oleoyldopamine. The endogenous antagonists of TRPV1 are lysozyme D2 and norepinephrine, while the exogenous antagonists include capsazepine iodine resin toxin, caffeic acid, SB-366791, AMG-9810, and A-425619 (Li et al., 2021). Use of TRPV1 agonists and antagonists along with TRPV1 receptor knockout mice has revealed a strong connection between TRPV1 and the development of fibrotic diseases (Hutter et al., 2005; Ueda et al., 2008; Sumioka et al., 2014; Wang Q et al., 2014). Table 1 lists the modulators available for evaluating TRPV1 association with fibrosis.

**TABLE 1:** Summary of the role played by TRPV1 in fibrotic disease.

Experimental model	Experimental setting	Treatment to modulate TRPV1 expression	Effects of treatment	Role of TRPV1 in fibrotic diseases	References
Myocardial fibrosis	*In vitro*	Capsaicin	Activation of TRPV1 attenuates angiotensin II-induced proliferation and differentiation of mouse cardiac fibroblasts	Beneficial	Wang Q et al. (2014)
Myocardial fibrosis	*In vivo*	Genetic deletion; dietary capsaicin	TRPV1 activation protected mitochondria from dysfunction by increasing cardiac mitochondrial sirtuin 3 expression, the proficiency of	Beneficial	Lang et al. (2015)
Renal fibrosis	*In vitro*	Genetic deletion	TRPV1 activation down-regulated TGFβ1/Smad2/3 signaling	Beneficial	Wang and Wang, (2011)
Pancreatic fibrosis	*In vitro*	Capsazepine	TRPV1 activation increased SP release, promoted plasma and protein extravasation to interstitial tissue and neutrophil infiltration	Detrimental	Hutter et al. (2005)
Corneal fibrosis	*In vitro*	Capsazepine	Activating TRPV1 expression promotes the proliferation and migration of mouse corneal epithelial cells and accelerates corneal fibrosis and inflammation	Detrimental	Sumioka et al. (2014)
Corneal fibrosis	*In vitro*	Capsazepine	Activation of TRPV1 leads to increased intracellular Ca levels, which promote the secretion of inflammatory mediators, including IL-6 and IL-8	Detrimental	Zhang et al. (2015)

## Correlation between TRPV1 and fibrosis

### TRPV1 and myocardial fibrosis

Cardiac fibrosis is a pathological change observed in the later stage of various cardiovascular diseases, usually manifesting as cardiac hypertrophy, ECM deposition, and collagen synthesis, which eventually lead to irreversible heart structure remodeling and cardiac dysfunction (González et al., 2018). Pathological cardiac remodeling subjects the heart to increased pressure and volume overload and affects all cardiac cells, including myocytes, endothelial cells, stromal cells, and cardiac fibroblasts (Heusch et al., 2014). Calcium ions regulate various cellular functions, and abnormal intracellular calcium homeostasis can activate fibrosis-associated remodeling. Calcium plays an important role in regulating apoptosis, mast gene expression, and myocardial fibrosis, while TRPV1 mediates Ca^2+^ signal transduction in cells (Ramires et al., 1998). Cardiac fibroblasts treated with the TRPV1 agonist capsaicin exhibit activation of TRPV1 channels followed by a significant increase in intracellular Ca^2+^ concentration (Wang Q et al., 2014). Intracellular Ca^2+^ entry facilitates fibroblast function (Inoue et al., 2006); for instance, the occurrence of myocardial fibrosis depends on cardiac fibroblast proliferation (Krenning et al., 2010). Specifically, TRPV1 overexpression substantially reduces isoproterenol-induced proliferation and differentiation in mouse cardiac fibroblasts and prevents the development of myocardial fibrosis (Wang et al., 2016). Furthermore, a high-salt diet can cause cardiac hypertrophy and fibrosis in mice whereas long-term capsaicin consumption can substantially decrease cardiac hypertrophy and fibrosis. After long-term capsaicin intervention, WT mice show reduction in high-salt-induced cardiac insufficiency and cardiac hypertrophy; however, these symptoms are not improved in TRPV1^-/-^ mice. Chronic dietary capsaicin intake can increase the proficiency of mitochondrial complex I OXPHOS, production of ATP, expression of cardiac mitochondrial sirtuin 3, and activity of complex I enzyme activity via activating TRPV1, leading to alleviation in cardiac mitochondrial dysfunction and prevention of cardiac hypertrophy induced by a high-salt diet (Lang et al., 2015). Ligation of the anterior descending branch of the left coronary artery is a common method to generate myocardial infarction models. This procedure damages myocardial contractility and aggravates concomitant development of left ventricular dilatation, myocardial fibrosis, and ventricular remodeling (Gao et al., 2010). Recently, capillary formation, myofibroblast deposition, and collagen expression in *TRPV1* ^-/-^ mice after ligation of the left anterior descending coronary artery were reported to be significantly higher than those in wild-type mice. This was accompanied by significant upregulation of vascular endothelial growth factors and matrix metalloproteinases, which exacerbated cardiac remodeling. Furthermore, *TRPV1* ^-/-^ mice displayed higher cardiac fibroblast infiltration, capillary density, and ECM collagen content. The affected processes markedly accelerated fibrosis and impaired myocardial contractility following myocardial infarction (Huang et al., 2010). Mice that received intrathecal resin toxin (TRPV1 agonist) showed reduced sympathetic nerve activity in an overactive heart and improved cardiac compliance. Resin toxin desensitizes cardiac afferent fibers by activating TRPV1 expression, thereby eliminating the sympathetic excitatory reflex of the heart, weakening cardiac remodeling, and alleviating cardiovascular dysfunction in rats with heart failure. Molecular evidence has demonstrated that resin toxin reduces cardiac hypertrophy, cardiac apoptosis, and fibrosis marker expression in rat models of chronic heart failure (Wang H.J et al., 2014; Wang et al., 2019). In addition, TRPV1 deficiency exacerbates myocardial hypertrophy and impairs diastolic function and myocardial interstitial fiber proliferation in a mouse model of transverse aortic constriction-induced *de novo* cardiac pressure overload (Zhong et al., 2018). After transverse aortic constriction (TAC) in TRPV1^-/-^ mice, secretion of pro-inflammatory cytokines as well as infiltration by macrophages increase, and cardiac function is deteriorated. Moreover, neuropeptide CGRP, which regulates vascular tone and cardiovascular function, cannot be released in the absence of TRPV1. Therefore, TRPV1 contributes to the anti-inflammatory process by inhibiting pressure overload-induced myocardial hypertrophy and exerting cardioprotective effects. However, results obtained in other studies represent another point of view. In a study using TAC to simulate acute pressure overload, the cardiac function of TRPV1^-/-^ mice was improved compared with that of wild type mice, and myocardial hypertrophy, fibrosis, and tissue remodeling were reduced (Buckley and Stokes, 2011). These findings suggest that TRPV1 knockout can preserve cardiac structure and function under simulated pressure overload. Further, the use of TRPV1 antagonists can prevent or improve myocardial hypertrophy and myocardial fibrosis and reduce the levels of apoptosis markers. Therefore, the TRPV1 receptor is a candidate therapeutic target for myocardial fibrosis. Notably, among other TRPV channels, knockout of the TRPV2 channel can effectively improve cardiac insufficiency and inhibit myocardial fibrosis (Entin-Meer et al., 2018). Activation of TRPV3 leads to interstitial fibrosis and aggravates cardiac dysfunction in pressure overloaded rats (Liu Y al., 2018) . TRPV4 gene deletion or siRNA-mediated downregulation is attenuated *in vitro* by TGF- β, and Trpv4^-/-^ mice show preserved myocardial integrity and cardiac function as well as reduced cardiac fibrosis (Adapala et al., 2020).

#### TRPV1 and renal fibrosis

Renal fibrosis is often associated with chronic kidney failure resulting from various causes, which eventually progresses to end-stage renal failure results in the destruction of kidney tissue structure and complete loss of kidney function (Humphreys, 2018). Hypertension-induced kidney injury elicits a fibrotic response via abnormal activation of renal fibroblasts and the overproduction of ECM proteins (Sun, 2019). Hypertension induced by deoxycorticosterone acetate salts (DOCA) causes glomerulosclerosis, increases the number of proliferating cell nuclear antigen (PCNA)-positive tubular cells, tubular interstitial fibrosis, and ECM over-deposition in both wild-type and *TRPV1* ^*-/-*^ mice; however, *TRPV1* ^-/-^ mice exhibit more severe symptoms than wild-type mice (Wang et al., 2008). *TRPV1* knockdown exacerbates renal damage and fibrosis induced by DOCA salt-mediated hypertension; thus, TRPV1 may be a part of a protective mechanism against hypertension-induced organ damage. Consistently, TRPV1 activation reportedly protects against renal damage during hypertension as TRPV1 loss promotes the upregulation of fibronectin, expression of type I and III collagen, which can delay the progression of interstitial renal fibrosis. (Wang and Wang, 2011). Furthermore, DOCA salt induces upregulation of SMAD2/3 phosphorylation and increases TGF-β1 activity. TGF-β1 protein expression in the kidneys of DOCA salt-induced hypertensive *TRPV1* ^*-/-*^ mice is significantly higher than that in WT mice. These results indicate that excessive damage to renal function and structure is accompanied by increased ECM protein production and TGF-β1/SMAD2/3 signaling pathway activation. Thus, interfering with TGF-β1 production may help reduce ECM accumulation and delay progression of renal interstitial fibrosis (Wang and Wang, 2009). Further, increased production of pro-inflammatory cytokines/chemokines, including TNF-α, IL-6 and MCP-1, was reported in the *TRPV1* ^-/-^ mouse model of DOCA-induced acute salt-sensitive hypertension. Thus, in the absence of TRPV1, many pro-inflammatory cytokines harmful to the kidney are produced along with increased macrophage infiltration and tubulointerstitial injury. In addition, abnormal activation of the nuclear transcription factor NF-κB as well as multiple inflammation-related genes causes excessive inflammatory responses that exacerbate renal damage and pathogenesis. The absence of TRPV1 produces a large number of pro-inflammatory cytokines harmful to the kidney, accompanied by increased macrophage infiltration and tubulointerstitial injury. In DOCA salt-treated WT mice compared with that in *TRPV1* ^*-/-*^ mice, it was obvious that NF-κB activity in *TRPV1* ^*-/-*^ mice-increased further. These data suggest that NF-κB plays a central regulatory role in mediating the anti-inflammatory and protective effects of TRPV1. In addition, abnormal activation of the nuclear transcription factor NF-κB as well as multiple inflammation-related genes induces excessive inflammatory responses that exacerbate renal damage and pathogenesis. NF-κB activity was obviously increased in TRPV1^-/-^ mice compared with that in DOCA salt-treated WT mice . These data suggest that NF-κB plays a central regulatory role in mediating the anti-inflammatory and protective effects of TRPV1 in renal tissues. Furthermore, available scientific evidence suggests that the TRPV1 receptor is expressed in human proximal tubular cells, mouse renal microvascular endothelial cells, canine renal tubular cells, and porcine renal tubular cells (Tsagogiorgas et al., 2012; Wang et al., 2017; Lu et al., 2020). *In vitro* studies have suggested that TRPV1 may be involved in renal fibrosis through the production of major pro-inflammatory mediators of apoptosis and can protect against renal fibrosis as a prospective regulator of inflammatory processes. Alternatively, *in vivo* studies using an ischemia/reperfusion model along with TRPV1 agonists (capsaicin and resin toxin) have shown that TRPV1 plays a role in the response to renal injury in mice. Moreover, TRPV1 agonists reportedly prevent ischemia/reperfusion-induced renal dysfunction and protect the kidneys from injury and fibrosis (Ueda et al., 2008; Chen et al., 2014). Thus, TRPV1 is involved in mediating inflammatory response and fibrosis in renal fibrosis, and its activation ameliorates both processes. The relationship between TRPV channels and renal fibrosis has received limited research attention. However, treatment with apigenin, an agonist of TRPV4, has been found to help alleviate the DOCA salt-induced damage to renal structure and function, reduce the expression of extracellular matrix proteins, and exert a beneficial effect against hypertension-induced renal fibrosis (Wei et al., 2017).

### TRPV1 and pancreatic fibrosis

Chronic pancreatitis is an irreversible and progressive inflammation of the pancreatic parenchyma and pancreatic ducts. Its pathological features present as chronic inflammatory damage and interstitial fibrosis of the pancreatic parenchyma, calcification of the pancreatic parenchyma, dilatation of the pancreatic ducts, and changes such as pancreatic duct stones (Barry, 2018). Pancreatic fibrosis, a characteristic feature of chronic pancreatitis, presents with the fibrosis of the pancreatic parenchyma, atrophy of lung alveolar cells, and infiltration of inflammatory cells (Huang et al., 2021). In pancreatitis, TRPV1 is activated in pancreatic sensory nerves and spinal cord neurons, thereby inducing the release SP and CGRP, causing pain and inflammation (Wick et al., 2006). SP plays pro-inflammatory role in acute pancreatitis and pancreatitis-related lung injury by binding to neurokinin 1 receptor (NK1R) and acts on calcium channels in cell membranes via inositol triphosphate, causing depolarization of membrane potential and alteration of protein kinase activity (Bhatia et al., 1998). Furthermore, in a mouse model of cerulein-induced pancreatitis, the use of the TRPV1 antagonist capsazepine inhibited primary sensory neurons and reduced tissue inflammation, thereby inhibiting the release of SP and the subsequent NK1R activation, eventually alleviating the severity of fibrosis in the pancreas (Nathan et al., 2001; Hutter et al., 2005). Alternatively, in a rat model of pancreatitis, CGRP administration induced vasodilation and vascular injury, which aggravated pancreatitis symptoms. Moreover, CGRP removes active digestive enzymes and increases the levels of inflammatory mediators in pancreatic tissue to reduce pancreatic injury (Warzecha et al., 2001). The severity of pancreatitis may be determined by the aggregation and activation of inflammatory cells after alveolar cell injury and the production and release of cytokines and other inflammatory mediators (Liddle and Nathan, 2004). TRPV1 expression is upregulated in pancreatic dorsal root ganglion neurons during chronic pancreatitis, and a significant reversal of pancreatic inflammation and pain relief was reported with systemic administration of the TRPV1 antagonist SB-366791 (Xu et al., 2007). Furthermore, another study showed that pancreatic inflammation combined with significant changes in the activity and function of TRPV1 increased the excitability of pancreatic sensory neurons entering the spinal cord as well as the vagus nerve (Schwartz et al., 2011). Based on these observations, we conclude that TRPV1 plays a key role in neuroinflammation-driven pancreatic fibrosis and blocking neurogenic inflammation may improve disease outcomes. However, this needs to be investigated and validated in pre-clinical and clinical models. In other studies on TRPV channels and pancreatic fibrosis, the TRPV4 antagonist hc067047 has been found to effectively alleviate pain-related behavior in the rat chronic pancreatitis model induced by alcohol/high fat diet (AHF) and reduce hypersensitivity and fibrosis pathology (Zhang et al., 2015). TRPV6 is a novel susceptibility gene for chronic pancreatitis. Administration of cerulein to *TRPV6* ^*mut/mut*^ mice leads to severe pancreatitis, characterized by elevated pancreatic enzyme levels, histological changes, and pancreatic fibrosis (Masamune et al., 2020).

#### TRPV1 and corneal fibrosis

Fibrosis of the corneal stroma, also known as scarring or progressive opacity, represents a disruption of corneal homeostasis following injury, resulting in abnormal tissue remodeling (Wilson et al., 2017). Excessive corneal fibrosis can form a scar in the center of the cornea, thereby reducing corneal transparency and causing visual impairments (Meek and Knupp, 2015). Recently, the role of TRPV1 in corneal fibrosis was explored *in vitro* and *in vivo*, and TRPV1 expression was observed in both corneal fibrotic tissue and epithelial cells. *TRPV1* deletion reportedly inhibited ocular fibroblast differentiation as well as expression of TGF-β1 and other pro-inflammatory genes, while TRPV1 suppression inhibited inflammation and fibrosis onset during corneal healing in mouse model of corneal alkali-burn (Okada et al., 2011). Myofibroblast activation promotes fibrosis, and persistent induction of fibrotic response results in fibrosis observed in many types of corneal trauma (Wilson, 2020). Corneal wound-induced myofibroblast differentiation is greatly inhibited by capsazepine (a TRPV1 antagonist) via downregulating α-SMA expression (Yang et al., 2013a). Release of endocannabinoids such as 2-AG following injury-induced cannabinoid receptor 1 (CB1) activation downregulates TRPV1-mediated inflammation and corneal opacity (Yang et al., 2013b). This finding suggests that inhibition of TRPV1 activation reduces corneal fibrosis and inflammation and CB1 is a candidate drug target for regulating TRPV1-induced inflammation. Suppressing TRPV1 ion channel expression or using TRPV1 antagonists are potential therapeutic strategies to reduce inflammation and enhance the healing of fibrotic wounds. Furthermore, TRPV1 activity is closely associated with an inward flow of cytosolic calcium (Yang et al., 2013c). Treatment of human corneal epithelial cells with capsaicin increases intracellular Ca^2+^ levels and increases IL-6 and IL-8 release by 3.3- and 9-fold, respectively. Thus, TRPV1 activation induces the secretion and release of inflammatory mediators from the human corneal epithelium which subsequently leads to corneal damage and fibrosis (Zhang et al., 2007). Furthermore, capsaicin (a TRPV1 agonist) promotes the proliferation and migration of corneal epithelial cells, whereas SB366791 (a TRPV1 antagonist) delays corneal fibrosis in mice. These results indicate TRPV1 activation induces the upregulation of IL-6 and SP expression, thereby delaying the healing of the corneal epithelium in mice (Sumioka et al., 2014). Collectively, these findings suggest that TRPV1 is an important player in mediating inflammation following *in vivo* injury and should be further explored as a therapeutic target for corneal fibrosis. In line with this, TRPV4 activation contributes to the severe inflammatory fibrosis caused by alkali damage to the cornea of mice. TRPV4 antagonists can improve corneal function recovery during wound healing by reducing inflammatory fibrosis (Okada et al., 2016).

### TRPV1 and pulmonary fibrosis

Pulmonary fibrosis is a chronic, interstitial lung disease characterized by thickening and scarring of the alveolar walls and is caused by long-term inflammation of the lungs, bacterial or viral infections, autoimmune reactions, and allergy and related tissue damage (Thannickal et al., 2004). Life expectancy after pulmonary fibrosis diagnosis is between 2 and 6 years (Selman et al., 2001). Pulmonary fibrosis is commonly characterized by coughing, difficulty in breathing, and fatigue (Swigris et al., 2005). However, few studies have examined the association between TRPV1 and pulmonary fibrosis. A recent study showed that bleomycin-induced pulmonary fibrosis is characterized by a positive correlation between neuropeptide SP, NK1R, and CGRP and cough sensitivity in animal models (Guan et al., 2021). Secretion of inflammatory factors and immune mediators indirectly activates the TRPV1 receptor. Furthermore, inflammation and the cough reflex pathway are markedly upregulated in pulmonary fibrosis, leading to increasing cough hypersensitivity and disease progression (Guo et al., 2019). Regarding bleomycin-induced pulmonary fibrosis, the symptoms of fibrosis are weaker in TRPV4^-/-^ mice than in wild type mice, and gene deletion of TRPV4 inhibits myofibroblast differentiation (Rahaman et al., 2014).

#### TRPV1-mediated pathways in fibrosis

Increasing evidence indicates that fibrotic diseases are associated with pathways such as those for inflammation, oxidative stress, the renin angiotensin system, and TGF- β/SMAD signaling (Chen et al., 2018; Otoupalova et al., 2020; Gupta et al., 2021, 2018). TGF-β1 receptor is one of the key elements associated with fibrosis and may play a critical role in infarct healing (Murphy-Ullrich and Suto, 2018). TGF-β1 is induced and activated in various fibrotic diseases, and it in turn induces its downstream mediators SMAD2 and SMAD3 to play their biological roles (Hu et al., 2018). In myocardial fibrosis, TRPV1-mediated angiotensin II-induced cardiac fibroblast differentiation inhibits ECM deposition via a downregulation-dependent TGF-β-SMAD signaling pathway (Huang et al., 2010; Wang H.J et al., 2014). Furthermore, TRPV1 activation upregulates TGF-β1 protein expression and can inhibit the TGF-β1 fibrogenic activity by downregulating SMAD2/3; these data suggest that TRPV1 inhibits the TGF- β/SMAD signaling pathway and plays a protective role in renal fibrosis induced by salt-sensitive hypertension (Wang and Wang, 2011). In pancreatic fibrosis, TRPV1 contributes to pain and fibrosis by regulating TGF-β expression via the SMAD pathway (Liu L et al., 2018). *TRPV1* silencing in corneal fibroblasts promotes TGF-β-induced corneal myofibroblast differentiation via SMAD2-p38 signaling, which also induces oxidative stress and accelerates corneal fibrosis progression (Yang et al., 2013a). Recent studies have elucidated a new signal transduction pathway leading to corneal fibrosis. The p38-MAPK pathway is involved in the migration and proliferation of cells during corneal epithelial tissue wound healing in mice. TGF-β1-activated p38-MAPK is one of the most important signaling cascades involved in cell migration (Saika et al., 2004). The limited information available regarding the regulatory role played by TRPV1 in fibrosis suggests that TRPV1 mediates fibrosis mainly via TGF-β signaling. Research studies exploring the other TRPV channels have focused on elucidating their relationship with TGF-β signaling (Rahaman et al., 2014; Liu Y et al., 2018; Kato et al., 2022). TRPV4 also protects the heart from myocardial infarction-induced pathological myocardial remodeling by regulating cardiac fibroblast differentiation and myocardial fibrosis through the Rho/MRTF-A signaling pathway (Adapala et al., 2020).

## Conclusion and prospects

In this review, we have comprehensively analyzed the role played by TRPV1 in fibrosis. Since TRPV1 channels are expressed in various organs, they play different roles in physiological and clinical conditions. TRPV1 mediates the entry of extracellular Ca^2+^ and promotes Ca^2+^ release from the endoplasmic reticulum into the cytoplasm, thereby regulating intracellular Ca^2+^ concentration and myofibroblast growth and migration. The neuropeptides CGRP and SP are released in conjunction with TRPV1 activation, causing neurogenic inflammation expressed as vasodilation, plasma extravasation, and podocyte enlargement. These neuropeptides promote leukocyte migration and regulate fibrosis progression in different organs. Furthermore, the TGF-β/SMAD signaling pathway is one of the promising targets to promote healing of fibrosis. Activation of TRPV1 favors renal fibrosis and inhibition of TRPV1 favors pancreatic fibrosis, corneal fibrosis and ocular corneal fibrosis. However, the contradictory role of TRPV1 in myocardial fibrosis makes it difficult to decide whether TRPV1 should be activated or inhibited when treating inflammation and fibrosis caused by organ damage. This phenomenon illuminates the complex role played by TRPV1 in pathophysiological conditions. Two experimental studies using TAC to simulate acute pressure overload reported contrasting results (Buckley and Stokes, 2011; Zhong et al., 2018). These contrasting results may be attributed to the different factors analyzed as well as different experimental conditions, such as different testing time points before and after cardiac echocardiography, differences in the size of the needles used to develop the TAC model, and different doses of antagonists used. The long-term efficacy and safety of the systemic interventions targeting TRPV1 remain unknown and may be influenced by post-inflammatory compensatory response. Although this makes understanding the role of TRPV1 and mechanisms underlying its function a challenge for developing anti-fibrotic therapy, TRPV1 is a potential therapeutic target to prevent fibrosis onset and development and ensuing organ damage. We previously showed that TRPV1 loss aggravates inflammation following myocardial infarction, impairs lesion healing, promotes myocardial fibrosis, and leads to poor ventricular remodeling (Wang and Wang, 2005). Nevertheless, TRPV1 may also protect the heart from injury by releasing the neurotransmitter SP, which acts as a cardiac anti-inflammatory molecule. Multiple studies have shown that TRPV1 activation can alleviate diabetes and cardiovascular diseases such as atherosclerosis and hypertension (Ma et al., 2011; Marshall et al., 2013). The neuropeptides released following TRPV1 activation exert multifaceted effects and regulate TRPV1 role in the inflammatory process at multiple levels. In conclusion, Ca^2+^, neuropeptides, and inflammation collectively modulate the role played by TRPV1 in fibrosis. Since little is known about the mechanisms underlying TRPV1-mediated fibrosis, further research to explore the role of TRPV1 in fibrosis is warranted. This will help in developing effective therapeutic interventions for fibrotic diseases in the future.
