# Supplementary material for: Transient receptor potential vanilloid subtype 1: A potential therapeutic target for fibrotic diseases
Source: Front Physiol. 2022 Aug 15;13:951980. doi: 10.3389/fphys.2022.951980 (PMC9420870; doi:10.3389/fphys.2022.951980)
Supplement: Supplementary file 1 [file Table1.DOCX]

**Table 1 Summary of the role played by TRPV1 in fibrotic disease**

| **Experimental model** | **Experimental setting** | **Treatment to modulate TRPV1 expression** | **Effects of treatment** | **Role of TRPV1 in fibrotic diseases** | **References** |
| --- | --- | --- | --- | --- | --- |
| Myocardial fibrosis | *In vitro* | Capsaicin | Activation of TRPV1 attenuates angiotensin II-induced proliferation and differentiation of mouse cardiac fibroblasts | Beneﬁcial | (Wang et al., 2014) |
| Myocardial fibrosis | *In vivo* | Genetic deletion; dietary capsaicin | TRPV1 activation protected mitochondria from dysfunction by increasing cardiac mitochondrial sirtuin 3 expression, the proficiency of | Beneﬁcial | (Lang et al., 2015) |
| Renal fibrosis | *In vitro* | Genetic deletion | TRPV1 activation down-regulated TGFβ1/Smad2/3 signaling | Beneﬁcial | (Wang et al., 2011) |
| Pancreatic fibrosis | *In vitro* | Capsazepine | TRPV1 activation increased SP release, promoted plasma and protein extravasation to interstitial tissue and neutrophil infiltration | Detrimental | (Hutter et al., 2005) |
| Corneal fibrosis | *In vitro* | Capsazepine | Activating TRPV1 expression promotes the proliferation and migration of mouse corneal epithelial cells and accelerates corneal fibrosis and inflammation | Detrimental | (Sumioka T et al., 2014) |
| Corneal fibrosis | *In vitro* | Capsazepine | Activation of TRPV1 leads to increased intracellular Ca levels, which promote the secretion of inflammatory mediators, including IL-6 and IL-8 | Detrimental | (zhang et al., 2011) |
